# 2-Acetylamino-3-[4-(2-acetylamino-2-carboxyethylsulfanylcarbonylamino) phenyl carbamoylsulfanyl] propionic acid, a glutathione reductase inhibitor, induces G_2_/M cell cycle arrest through generation of thiol oxidative stress in human esophageal cancer cells

**DOI:** 10.18632/oncotarget.18705

**Published:** 2017-06-27

**Authors:** Xia Li, Zhiming Jiang, Jianguo Feng, Xiaoying Zhang, Junzhou Wu, Wei Chen

**Affiliations:** ^1^ Zhejiang Cancer Research Institute, Zhejiang Cancer Hospital, Zhejiang Cancer Center, Hangzhou, Zhejiang 310022, China; ^2^ Zhejiang Key Laboratory of Diagnosis and Treatment Technology on Thoracic Oncology (Lung and Esophagus), Zhejiang Cancer Hospital, Hangzhou, Zhejiang 310022, China; ^3^ Zhejiang Key Laboratory of Radiation Oncology, Zhejiang Cancer Hospital, Hangzhou, Zhejiang 310022, China; ^4^ ACEA Bio CO., Ltd., Hangzhou, Zhejiang 310030, China

**Keywords:** glutathione reductase, oxidative stress, *S*-glutathionylation, cell cycle arrest, microtubule depolymerization

## Abstract

Esophageal squamous cell carcinoma (ESCC) is a highly malignant cancer with poor response to both of chemotherapy and radiotherapy. 2-Acetylamino-3-[4-(2-acetylamino-2-carboxyethylsulfanylcarbonylamino) phenyl carbamoylsulfanyl] propionic acid (2-AAPA), an irreversible inhibitor of glutathione reductase (GR), is able to induce intracellular oxidative stress, and has shown anticancer activity in many cancer cell lines. In this study, we investigated the effects of 2-AAPA on the cell proliferation, cell cycle and apoptosis and aimed to explore its mechanism of action in human esophageal cancer TE-13 cells. It was found that 2-AAPA inhibited growth of ESCC cells in a dose-dependent manner and it did not deplete reduced glutathione (GSH), but significantly increased the oxidized form glutathione (GSSG), resulting in decreased GSH/GSSG ratio. In consequence, significant reactive oxygen species (ROS) production was observed. The flow cytometric analysis revealed that 2-AAPA inhibited growth of esophageal cancer cells through arresting cell cycle in G_2_/M phase, but apoptosis-independent mechanism. The G_2_/M arrest was partially contributed by down-regulation of protein expression of Cdc-25c and up-regulation of phosphorylated Cdc-2 (Tyr15), Cyclin B1 (Ser147) and p53. Meanwhile, 2-AAPA-induced thiol oxidative stress led to increased protein *S*-glutathionylation, which resulted in α-tubulin *S*-glutathionylation-dependent depolymerization of microtubule in the TE-13 cells. In conclusion, we identified that 2-AAPA as an effective thiol oxidative stress inducer and proliferation of TE-13 cells were suppressed by G_2_/M phase cell cycle arrest, mainly, through α-tubulin *S*-glutathionylation-mediated microtubule depolymerization. Our results may introduce new target and approach for esophageal cancer therapy through generation of GR-mediated thiol oxidative stress.

## INTRODUCTION

Esophageal squamous cell carcinoma (ESCC), the predominant type of esophageal cancer in Asia [1, 2], is a malignancy associated with high mortality [3, 4]. In China, esophageal cancer is the fourth most frequently diagnosed cancer and the fourth leading cause of cancer death [5]. Multidisciplinary treatment including surgery, radiotherapy and chemotherapy is generally approached to treat locally advanced and metastatic ESCC [3]. Unfortunately, owing to its aggressive nature and poor response to chemotherapy and radiotherapy, although adjuvant chemo- and radio-therapy have resulted in relieving symptoms and improving the life quality of patients with esophageal cancer, the overall 5-year survival rate for all patients with esophageal cancer is less than 20% [6–8], therefore esophageal cancer still remains a challenging cancer disease to treat [9, 10]. Exploring more effective therapeutic agents and approaches to treat esophageal cancer is urgently needed.

Oxidative stress plays an important role in regulation of cancer cell behavior [11, 12]. Its induction is known associated with reactive oxygen species (ROS) production. ROS are intracellular chemical species and uncontrolled elevation of ROS can lead to direct damage of lipids, proteins and DNA [13, 14]. ROS include the superoxide anion (O_2_^-^), hydrogen peroxide (H_2_O_2_), as well as hydroxyl radicals (OH· ) [14, 15]. Induction of oxidative stress and production of ROS are known to increase the rate of mutations in the cells [11]. The role of ROS in tumorigenesis is contradictory. Low level of ROS promotes cancer initiation, progression and involves in the conduction of signaling pathways that regulate cancer cell proliferation, survival, angiogenesis and metastasis [14]. Cancer cells have a higher level of oxidative stress than non-malignant cells, thus they are vulnerable to the acute induction of oxidative stress caused by agents inducing ROS [11]. Therefore, excessive levels of ROS in cancer cells can lead to cell death and cell cycle arrest [14, 16]. ROS also can exert signaling functions by modulating, at different layers, protein oxidation since proteins have “cysteine switches” that can be reversibly reduced or oxidized, supporting the dynamic signaling regulation function. In this scenario, *S*-glutathionylation is a posttranslational modification involved in oxidative cellular response [17]. The glutathionylation/deglutathionylation cycle of cysteine residues depends on the redox state of the microenvironment, and reversible *S*-glutathionylation may help protect proteins from irreversible thiol oxidation reactions as well as providing a mechanism to transfer redox information [18–20].

2-Acetylamino-3-[4-(2-acetylamino-2-carboxyethylsulfanylcarbonylamino) phenyl carbamoylsulfanyl] propionic acid (2-AAPA) is a novel irreversible inhibitor of GR developed earlier and it is more potent than the most commonly used irreversible GR inhibitor BCNU [21]. Previously, 2-AAPA has been found to induce cellular thiol oxidative stress in the monkey kidney CV-1 cells and human ovarian cancer OVCAR-3 cells [22–24]. Induction of apoptosis and cell cycle arrest by 2-AAPA also has been observed in melanoma cells [25]. However, the effects of 2-AAPA in esophageal squamous cells are still unknown. The present studies were aimed to explore the effects of 2-AAPA on proliferation of TE-13 cells and the possible mechanism of act in 2-AAPA-induced G_2_/M phase cell cycle arrest.

## RESULTS

### The cytotoxicity of 2-AAPA in ESCC cells

**Figure 1:**
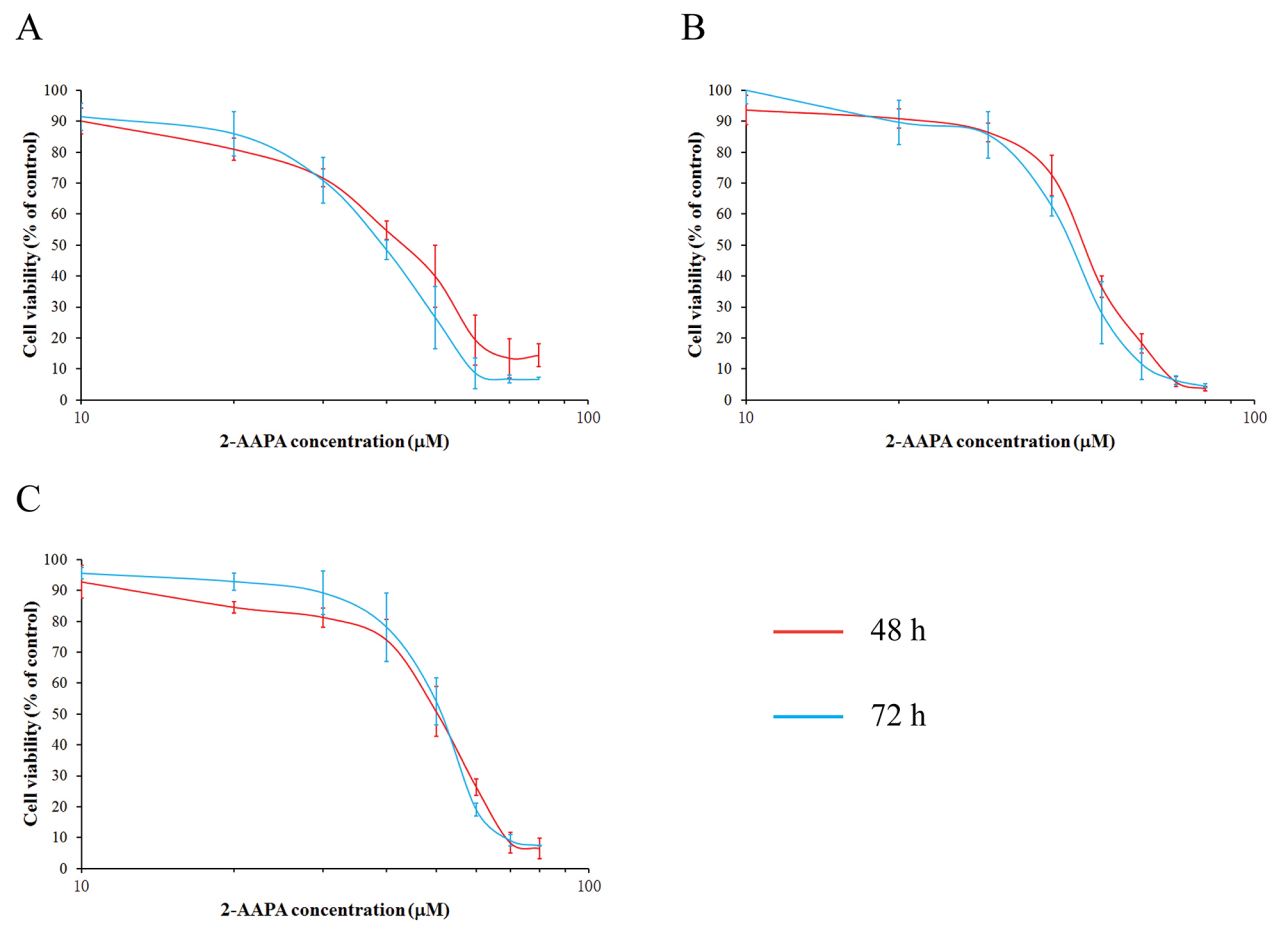
2-AAPA induced cytotoxicity in ESCC cells The ESCC cells (**(A)**, TE-13; **(B)**, KYSE-450; **(C)**, KYSE-510) were treated with various concentrations of 2-AAPA for 2 or 3 days. Cell survival rates were determined by the MTT assay. The data are presented as the mean ± SD of three independent experiments.

As previously reported, 2-AAPA exhibited inhibition of cell growth in various cancer cells [25]. To confirm the cytotoxicity of 2-AAPA in ESCC cells, its effects were examined on the cell viability of TE-13, KYSE-450 and KYSE-510 cells. As shown in Figure 1, 2-AAPA induced very similar cytotoxicity in these three ESCC cell lines, and its IC_50_s against TE-13, KYSE-450 and KYSE-510 cells were determined to be 46.43±6.84 μM, 47.42±1.49 μM and 52.90±1.45 μM for 48 h treatment, 42.52±4.40 μM, 43.24±2.24 μM and 50.56±2.06 μM for 72 h treatment, respectively. 2-AAPA effectively suppressed growth of ESCC cells *in vitro*.

### Intracellular GR activity reduction and thiol oxidative stress generation induced by 2-AAPA

**Figure 2:**
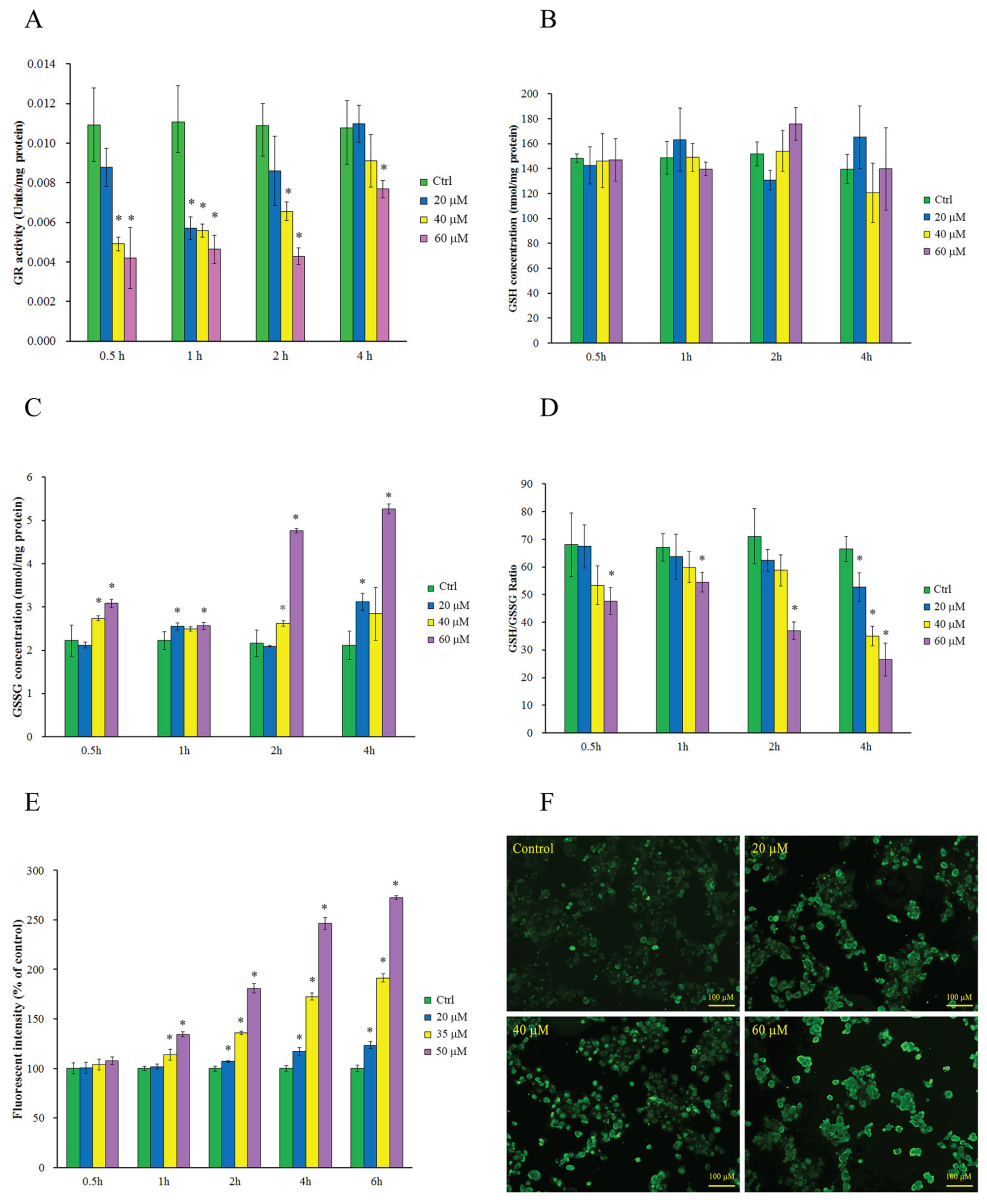
Intracellular GR activity reduction, thiol oxidative stress generation and protein *S*-glutathionylation induced by 2-AAPA **(A)** GR activity in the control and 2-AAPA-treated TE-13 cells. The data are presented as unit/mg protein. **(B, C & D)** Evaluation of intracellular GSH, GSSG, and GSH/GSSG ratio in the control and 2-AAPA-treated TE-13 cells. **(E)** ROS production in the control and 2-AAPA-treated TE-13 cells. The data are presented as fluorescence intensity. **(F)** 2-AAPA increased protein *S*-glutathionylation in TE-13 cells. The cells were treated with the indicated concentrations of 2-AAPA for 1 h, followed by fixation, and fluorescent staining as described in Materials and Methods. The data are derived from one of the three independent experiments. Cells were viewed under a fluorescent microscope. *, P < 0.05 compared with control group. All the data are expressed as the mean ± SD of three independent experiments.

GR is responsible for maintaining the supply of reduced glutathione, one of the most abundant reducing thiols in the majority of cells. In its reduced form, GSH plays key roles in the cellular control of reactive oxygen species [26]. The GR inhibitory effects of 2-AAPA were determined in TE-13 cells over a 4 h period. The results shown in the Figure 2A indicated that the maximum GR inhibition was observed within 1 h in the cells treated with 2-AAPA. The maximum inhibition was determined to be 48.5%, 55.1%, 61.7% compared to the control cells at concentrations of 20, 40 and 60 μM, respectively. The inhibitory effects induced by higher concentrations of 2-AAPA lasted longer than lower ones. The GR activity started to return after 1 h and almost recovered at 4 h except the high concentration samples.

To determine the extent consequences of GR inhibition induced by 2-AAPA, intracellular GSH and GSSG in TE-13 cells were quantified. As shown in Figure 2B, no significant depletion of GSH was observed in all the 2-AAPA-treated samples, however, substantial increase in GSSG (Figure 2C) was observed in 2-AAPA-treated cells. 0.15 to 1.5 fold increase of GSSG was observed in the TE-13 cells treated with 60 μM of 2-AAPA in 0.5 h to 4 h period. The intracellular GSH/GSSG ratios, a major index reflecting intracellular thiol oxidative stress, were calculated and presented in Figure 2D. In the control cells, the average GSH/GSSG ratio was determined to be 67.5/1. In 2-AAPA-treated TE-13 cells, the GSH/GSSG ratios were significantly decreased to be in the range of 70.3/1–26.6/1 depending on concentration and incubation time studied. These observations indicated that 2-AAPA rapidly decreased GR activity and changed the intracellular GSH/GSSG ratio.

Glutathione is one of the most important antioxidants in the cells. In its reduced form, GSH is capable of scavenging reactive oxygen species, thereby contributing to the control of redox homoeostasis [26]. Therefore, inhibition of GR by 2-AAPA produced ROS and increased intracellular thiol oxidative stress. The ROS production in the 2-AAPA-treated TE-13 cells is shown in Figure 2E. No significant difference in production of ROS was noticed between the control and treated cells in the first time point investigated. ROS was keeping accumulating during the experiment time period that made treated cells significantly different to the control. These results suggested that 2-AAPA-induced ROS generation was time- and dose- dependent.

Exposure to ROS is causatively linked to the disease pathologies associated with redox imbalance. In particular, ROS can differentially oxidize certain cysteine residues in target proteins and the reversible process of *S*-glutathionylation may mitigate or mediate the damage [27]. The global protein *S*-glutathionylation in TE-13 cells induced by 2-AAPA was visualized using an *S*-glutathionylated protein detection kit via fluorescent microscopy. As shown in Figure 2F, 2-AAPA induced significant increase of protein *S*-glutathionylation in 1 h-treatment in a concentration-dependent manner.

### The effects of 2-AAPA on cell cycle and apoptosis

**Figure 3:**
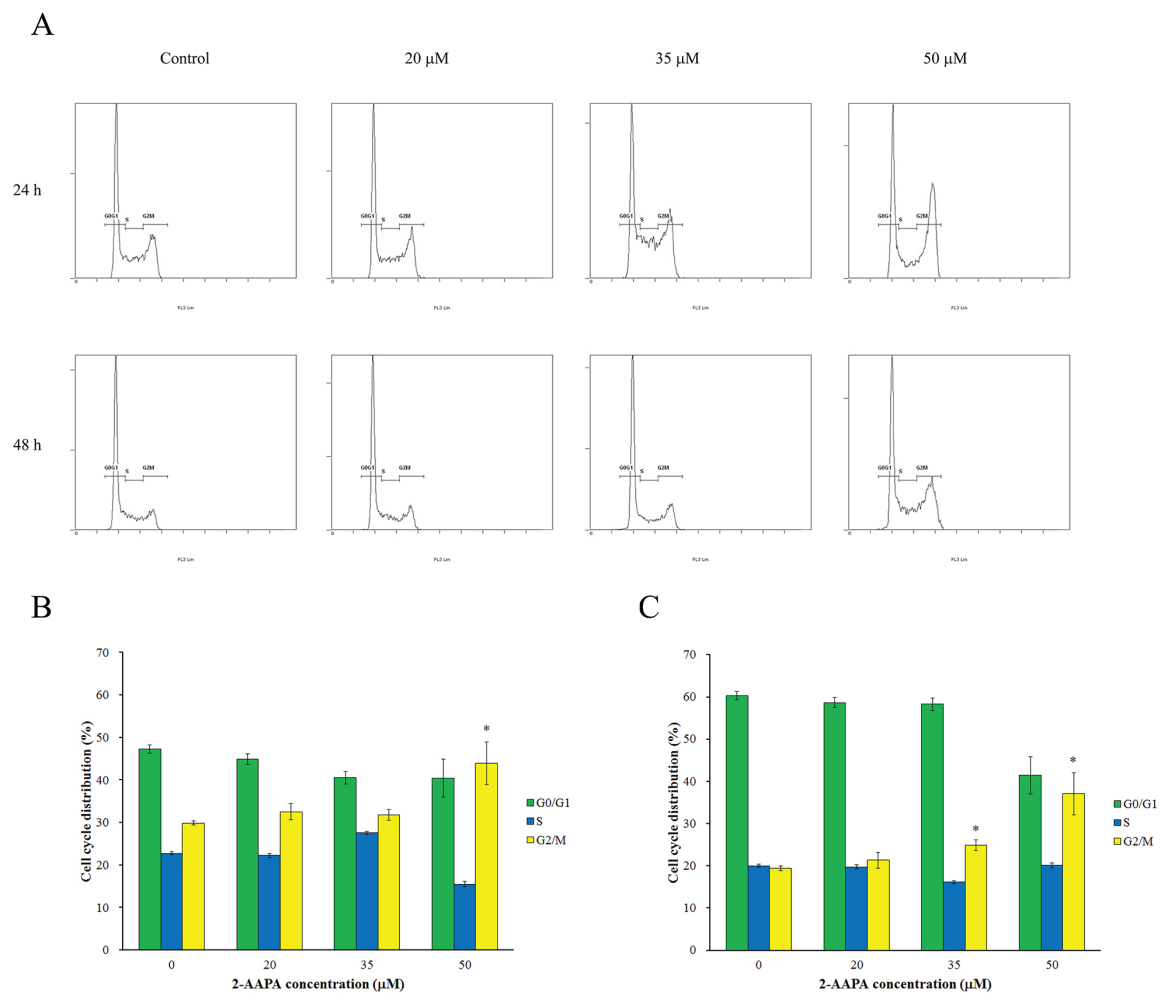
Effect of 2-AAPA on cell cycle distribution in TE-13 cells **(A)** The histograms of the TE-13 cells treated with various concentrations of 2-AAPA for 24 h and 48 h are presented. **(B & C)** The bar presentations reflects the quantification of cell cycle distribution at 24 h and 48 h, respectively. Results are presented as the mean ± SD of three independent experiments. *, P < 0.05 indicates statistical significance in 2-AAPA treated groups as compared to the control.

In order to understand the cell growth inhibition induced by 2-AAPA, the cell cycle distribution in TE-13 cells was investigated by flow cytometry. As shown in Figure 3, 2-AAPA at 20 μM produced small changes in DNA content between all phases of cell cycle. Whereas, 50 μM of 2-AAPA at 24 h-treatment resulted in significantly (P < 0.05) G_2_/M phase cell cycle arrest compared to the control. In 48 h treatment, 2-AAPA at 35 μM and 50 μM concentrations both induced significant cell cycle arrest in G_2_/M phase. The increase in cell population in G_2_/M phase was associated with a corresponding decrease in the population of cells mainly in G_0_/G_1_ phase.

**Figure 4:**
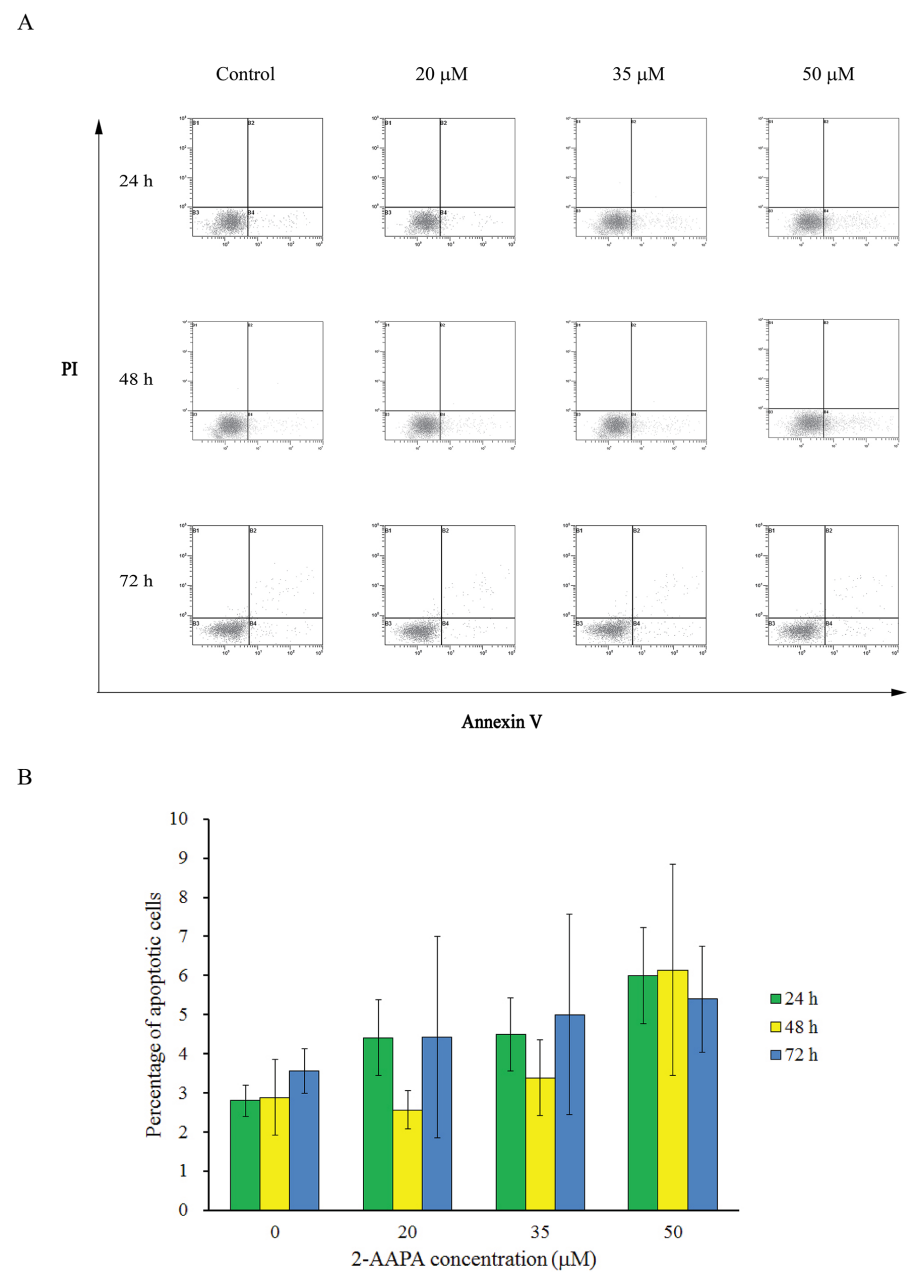
Effect of 2-AAPA on the induction of apoptosis in TE-13 cells TE-13 cells were treated with different concentrations of 2-AAPA for 24 h, 48 h and 72 h and then stained with Annexin V and PI. **(A)** Apoptotic assays were performed by flow cytometry. **(B)** The percentages of apoptotic cells are presented for 24 h, 48 h and 72 h treatments. Results are presented as the mean ± SD of three independent experiments.

Induction of cell cycle arrest and apoptosis is an important criterion to evaluate the therapeutic response of anticancer agents. Flow cytometric analysis was performed to investigate the effects of 2-AAPA on cell cycle and apoptosis in TE-13 cells. As shown in Figure 4, the highest concentration of 2-AAPA (50 μM) only induced 3.2%, 3.3% and 1.8% increase of apoptotic cells compared to the control in 24 h, 48 h and 72 h treatments, respectively. These observations thus strengthen our findings that 2-AAPA inhibits ESCC cells viability through a G_2_/M phase cell cycle arrest but apoptosis-independent manner.

### Identification of cell cycle regulatory proteins potentially targeted by 2-AAPA in TE-13 cells

**Figure 5:**
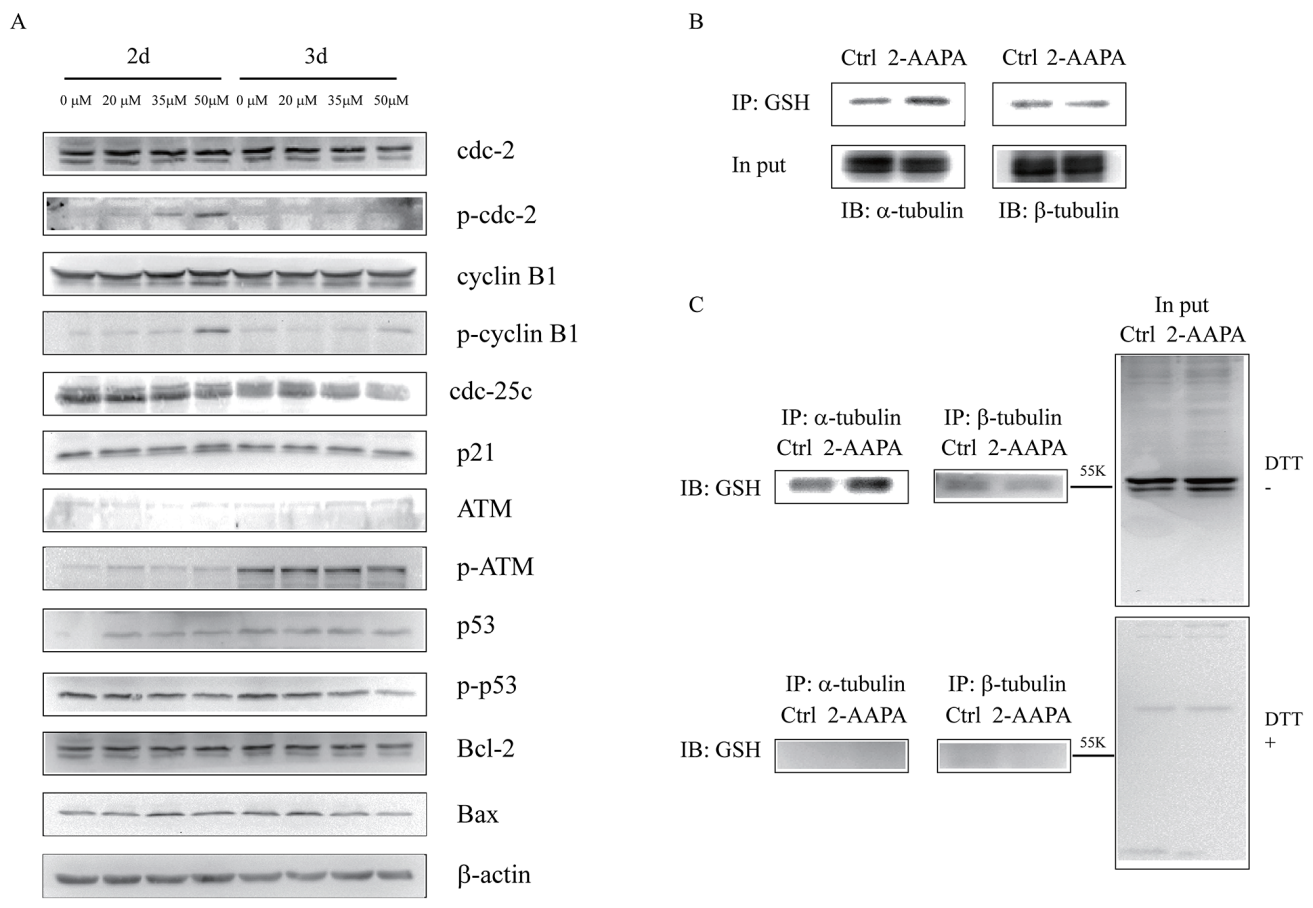
Identification of proteins potentially regulated by 2-AAPA in TE-13 cells **(A)** Assessment of the possible signaling pathways involved in G_2_/M phase cell cycle arrest and apoptosis. TE-13 cells were treated with 2-AAPA at the indicated concentrations for 48 h and 72 h, followed by the Western blot analysis for detection of Cdc-2, Cdc-25c, p-Cdc-2, Cyclin B1, p-Cyclin B1, p21, ATM, p-ATM, p53, p-p53, Bcl-2 and Bax expressions. β-actin was used as a loading control. The data are derived from one of the three independent experiments. **(B and C)** TE-13 cells were treated the same as in (A). Protein *S*-glutathionyaltion on α/β-tubulin was examined by reciprocal immunmoprecipitation and western blotting analysis as indicated.

To delineate the molecular mechanism of 2-AAPA-induced G_2_/M phase arrest, the expression of G_2_/M phase-associated proteins was evaluated in TE-13 cells after exposure to 20, 35 and 50 μM of 2-AAPA for 2 d and 3 d. The results (Figure 5A) indicated that 2-AAPA treatment decrease the expression of Cdc-25c and increase the expression of phosphorylated Cdc-2 (Tyr15) and Cyclin B1 (Ser147). The expression of p53 increased in 2 d-treatment but remained no change in 3 d-treatment. 2-AAPA showed no effect on protein expression of Cdc-2, Cyclin B1, p21, p-ATM (Ser1981), p-p53 (Ser15). In addition, 2-AAPA did not change the protein level of Bcl-2 and Bax, which contribute to apoptosis.

### 2-AAPA induces microtubule depolymerization in TE-13 cells

**Figure 6:**
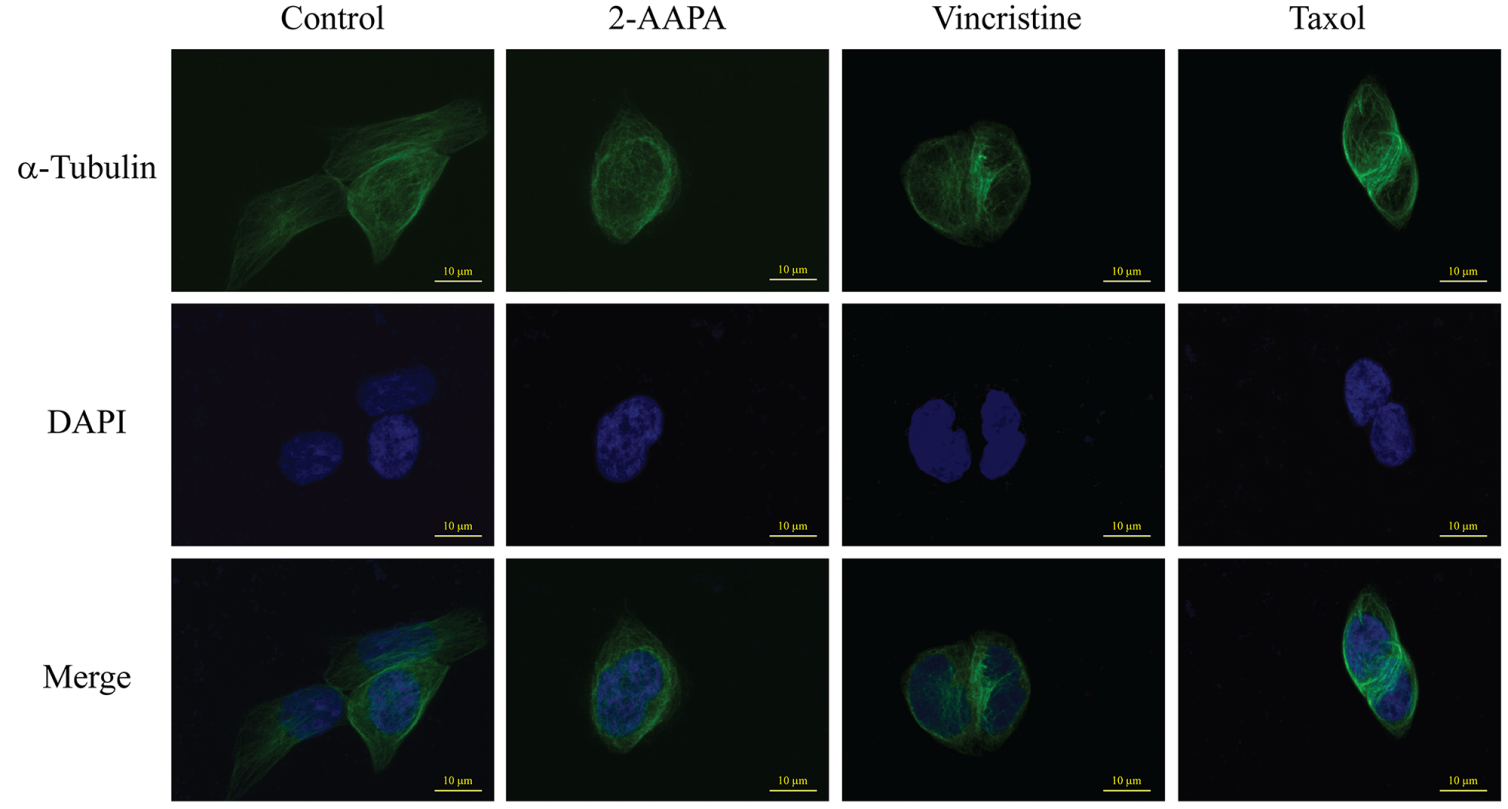
2-AAPA induces microtubule depolymerization in TE-13 cells TE-13 cells were treated with the indicated concentrations of 2-AAPA for 5 h, followed by fixation, permeabilization and indirect immunofluorescent analysis with an anti-α-tubulin-FITC. Nuclei were stained with DAPI. Taxol and vincristine sulfate were employed as positive controls for microtubule stabilization and microtubule depolymerization, respectively. Fluorescent images were captured by an Olympus Fluoview FV1200 microscope. The data are derived from one of the three independent experiments.

Microtubules is one of the central components involved in cell mitosis, thus the structure of microtubules were investigated by the incorporation of a fluorescent marker into microtubules. As shown in Figure 6, microtubules formed an intact network with fine filaments in the untreated cells, while a significant reduction of microtubule polymer mass was observed in 2-AAPA-treated cells. To compare the effects with that of vincristine sulfate, a microtubule depolymerizing antimitotic drug, and that of taxol, a microtubule stabilizing antimitotic drug, the same experiments were conducted with vincristine and taxol. The results showed that taxol induced condensation of microtubules polymer, while the effects induced by 2-AAPA was quite similar to that of vincristine sulfate, revealing that 2-AAPA depolymerized microtubules.

In order to examine the mechanism by which 2-AAPA induced microtubule depolymerization, the *S*-glutathionylation on tubulins were examined via reciprocal immunoprecipitation. As shown in Figure 5B, immunoprecipitation analysis indicated that *S*-glutathionylation on α-tubulin was elevated by 2-AAPA treatment in the TE-13 cells, while that of β-tubulin remained no change.

### Cell morphological change induced by 2-AAPA

**Figure 7:**
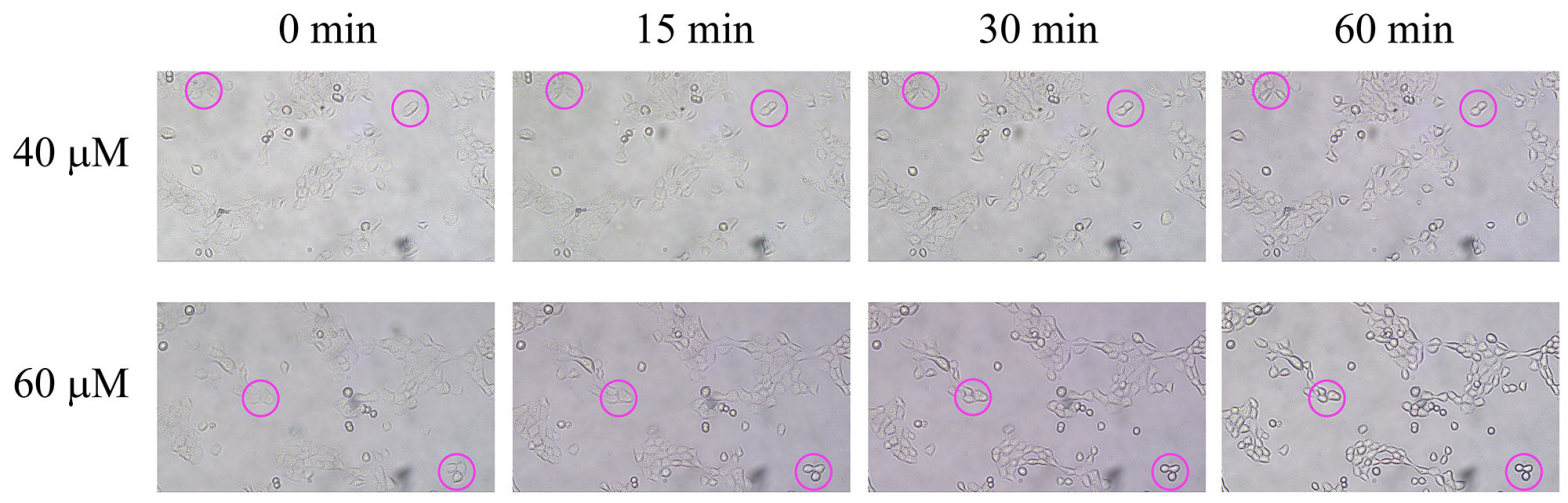
Effect of 2-AAPA on cell morphology in TE-13 cells TE-13 cells were treated with 40 and 60 μM of 2-AAPA. Some of the cells undergoing morphological changes are marked by red circles.

As one of the consequences of microtubule depolymerization, the morphology of the cells was found to be changed. The cell morphological changes were observed as early as 15 min and almost all the TE-13 cells treated with 60 μM of 2-AAPA shrank and detached from the culture dish at the end of 60 min (Figure 7). As comparison, 40 μM of 2-AAPA induced moderate morphological changes in the TE-13 cells.

## DISCUSSION

2-AAPA, a GR inhibitor, has been reported to be able to produce intracellular ROS and generate thiol oxidative stress in various cells [21–23, 25]. In this study, we investigated the growth inhibitory effects of 2-AAPA on proliferation of human ESCC cells *in vitro* and its possible mechanism of action. The results indicated that 2-AAPA exerted cytotoxic effect on TE-13 cells through generation of thiol oxidative stress, followed by ROS production (Figure 2E) leading to G_2_/M cell cycle arrest (Figure 3). The intracellular GR activity assay showed that 2-AAPA rapidly and significantly suppressed the GR activity in the TE-13 cells (Figure 2A). The GR activity started to return after 1 h. As reported earlier that 2-AAPA is an irreversible inhibitor of GR [21], so the activity of 2-AAPA-modified GR is unable to recover. Therefore, the returned GR activity may be contributed by new synthesized GR. As a result of GR inhibition, the intracellular GSSG concentration significantly increased but GSH level did not change (Figure 2B & 2C). Moreover, the ratio of GSH/GSSG, an indicator of thiol oxidative stress, decreased significantly (Figure 2D) reflecting the generation of thiol oxidative stress induced by destroying intracellular thiol homeostasis.

Redox regulation involves a variety of complex signaling networks where the concentrations of cellular thiols and ROS appear to play a pivotal role in determining whether cells undergo growth or apoptosis. How these signals dictate cell death or salvation is still unclear. Whilst low doses of ROS cause cell proliferation and stimulate growth, high concentrations induce temporary growth arrest, apoptosis and necrosis [26, 28–31]. Current study revealed that there are increased ROS levels in 2-AAPA-treated TE-13 cells compared with untreated cells in a dose-dependent manner. The results revealed that 2-AAPA is very effective in inducing ROS in TE-13 cells. Accumulating evidence indicates that many types of cancer cells exhibit elevated levels of ROS [32–38]. Moderate and controlled increase in ROS is necessary for cell proliferation and differentiation. Whereas excessive levels of ROS can be toxic to the cells, the anticancer drug-induced ROS stress can cause more damage in cancer cells than in normal cells and trigger cell death [39–43]. Thus, induction of ROS is potential therapeutic approach to selectively kill cancer cells without causing significant toxicity to normal cells. Among intracellular antioxidant molecules, GSH is the most abundant intracellular non-protein thiol in cells [44, 45], and it is considered as the major regulator of the intracellular redox state and participates in redox reactions via reversible oxidation of its active thiol. Upon oxidative stress, GSH is oxidized to its oxidized form GSSG, which is reduced back to GSH by the enzyme GR. Therefore, GR is critical for in maintaining the ratio of GSH/GSSG and protecting cells against oxidative stress. This study has shown that inhibition of GR activity is an effective way to generation of oxidative stress and suppression of cell growth in esophageal cancer cells.

As reported by Chen et al., 2-AAPA induced cell cycle arrest at G_2_/M phase and apoptosis in melanoma cells [25]. In addition, researches also showed that ROS-generating agents induced cell cycle arrest in accompany with apoptosis [46, 47]. Interestingly, 2-AAPA only induced significant cell cycle arrest in G_2_/M phase without significant apoptosis induction in TE-13 cells (Figures 3 & 4). P53, a tumor suppressor, is a potent transcription factor that, in response to a variety of cellular stresses, including DNA damage, oxidative stress, chemotherapeutic drugs and many aberrant growth signals. P53 controls the expression of a wide array of genes involved in cell cycle control, DNA repair, and apoptosis [48]. In addition, p53 controls G_2_ checkpoints and expression of wild-type p53 can prevent G_2_/M transition in mouse and human cell lines [49–55]. Following activation by cellular stress, p53 induces the expression of the cdk inhibitor p21, which is induced and localized to the nucleus in p53 wild-type cells undergoing G_1_ arrest, but not in cells expressing mutant p53 [56, 57]. Progression of cells from G_2_ to M phase is known regulated by Cdc-2/Cyclin B complexes, which have been identified as a principal component of the mitosis-promoting factor (MPF) [58, 59]. Cdc-25c, a phosphatase that activates Cdc-2 by dephosphorylating it at Tyr15, has been shown to be a target of repression by p53 [60]. Our data suggested that, in 2 d-treatment, 2-AAPA upregulated the expression of p53, which in turn decreased the protein of Cdc-25C. And then down-regulation of Cdc-25C increased inactivation form of phosphorylated Cdc-2. Eventually, the accumulation of inactive Cdc-2/Cyclin B1 complex led to G_2_ arrest [61]. Other key factors modulating cell cycle arrest including p21 and ATM showed no significant changes in the 2-AAPA-treated TE-13 cells. Therefore, 2-AAPA may directly activate p53, which is predicted to bind to and repress Cdc-25c, preventing Cdc-25c from dephosphorylating Cdc2 and initiating the transition from G_2_ to M phase.

Protein *S*-glutathionylation consists in glutathione reaction with the free thiol (SH) in certain cysteine (Cys) residues of proteins and it has been proposed as a reversible process of storing GSH during oxidative stress, and has been regarded as a protective mechanism against irreversible protein thiol-oxidation [62, 63]. Studies have shown that many proteins can be *S*-glutathionylated or deglutathionylated during oxidative stress [64–66] and in this study we demonstrate that the *S*-glutathionylation of proteins also present during 2-AAPA treatment. In agreement with our findings, previously demonstrated that in some cancer cell lines proliferation inhibitors rapidly induce a substantial increase of protein *S*-glutathionylation levels [67, 68]. 2-AAPA significantly increased protein *S*-glutathionylation in the TE-13 cells (Figure 2F). The anti-GSH antibody detected protein *S*-glutathionylation mainly located around 55 KD region, where tubulins appear (Figure 5C). 2-AAPA induced cell morphological changes in the TE-13 cells (Figure 7), which is very similar as observation in melanoma cells [25]. In addition, microtubules were found to be depolymerized in the TE-13 cells treated with 2-AAPA (Figure 6). As malfunction of microtubules can lead to in cell mitosis arrest and cell morphological changes (Figure 7), further reciprocal immunoprecipitation using anti-GSH and anti-tubulin (α and β) antibodies was performed and the results showed that the *S*-glutathionylation increased in α-tubulin but not β-tubulin by 2-AAPA treatment. In view of the critical role of the thiol functional groups in microtubule polymerization, it is reasonable to conclude that 2-AAPA increased protein *S*-glutathionylation in α-tubulin leading to microtubule depolymerization followed by G_2_/M phase cell arrest. As we mentioned earlier, 2-AAPA elevated GSSG and ROS level in TE-13 cells, thus the ROS-dependent *S*-glutathionylation may produce via SH/SS exchange reaction spontaneously [69]. In addition, 2-AAPA was found to be an inhibitor of glutaredoxin (Grx) [70], the enzyme catalyzes reduction of *S*-glutathionylated protein (protein-SSG). Taken these together, *S*-glutathionylation can be significantly generated and maintained in the TE-13 cells by 2-AAPA. Further studies are required to identify the specific *S*-glutathionylation site(s), which could be a potential target in regulation of microtubule dynamics.

In conclusion, it was found that 2-AAPA has the capability of inducing intracellular thiol oxidative stress in the TE-13 cells by GR inhibition and suppressing growth of ESCC cells. The cell proliferation inhibition induced by 2-AAPA was mainly through G_2_/M phase cell arrest but not apoptosis. The increased expression of p53 and phosphorylation of Cdc-2 (Tyr15) and Cyclin B1 (Ser147) which involved in G_2_/M phase regulation was observed. Moreover, microtubule depolymerization was detected in the 2-AAPA-treated TE-13 cells and it was caused by increased *S*-glutathionylation specifically in α-tubulin. The results suggested that 2-AAPA is a promising anti-esophageal cancer agent by producing intracellular thiol oxidative stress which is an efficient way inhibiting cancer cell growth. GR, as one of the key enzymes regulating cellular redox homoeostasis, can be a potential target in treating esophageal cancer.

## MATERIALS AND METHODS

### Materials

2-AAPA was synthesized in this laboratory according to the previously published method and prepared as a 100 mM stock solution in DMSO for cell based assays. Fetal bovine serum (FBS), RPMI 1640 growth medium, phosphate buffered saline (PBS), penicillin/streptomycin solution and 0.25% trypsin-EDTA solution were purchased from Gibco (Grand Island, NY, USA). ROS kit was from Beyotime (Haimen, Jiangsu, China). *S*-glutathionylated protein detection kit was from Cayman Chemical Company (MI, USA). BD Pharmingen™ FITC-Annexin V Apoptosis Detection Kit I was from BD Biosciences (San Jose, CA, USA). Primary antibodies against a-tubulin, b-tubulin, Cyclin B1, p21, p53, Cdc-25c, Cdc-2 and ATM were from Proteintech (Hubei, China). p-p53, p-Cdc-2, p-Cyclin B1 were from Santa Cruz Biotechnology (CA, USA). b-actin, Bcl-2 and Bax were from Cell Signaling Technology, Inc. (MA, USA). p-ATM was from Abcom (MA, USA). Pierce® ECL Plus kit was purchased from Lumigen, Inc. (Southfield, MI, USA). Other reagents was obtained in their highest purity grade available commercially.

### Cell line and culture conditions

Human esophageal cancer cell lines TE-13, KYSE-450 and KYSE-510 were maintained in this laboratory. The cells were cultured at 37°C in a humidified atmosphere of 5% CO_2_ in RPMI-1640 medium supplemented with 10% FBS, 100 U/mL of penicillin and 100 μg/mL of streptomycin.

### Cell viability assay

2-AAPA was tested *in vitro* for cytotoxicity against esophageal cancer cell lines TE-13, KYSE-450 and KYSE-510 in 96-well plates using the MTT assay to determine the cell viability. In brief, cells at a density of 4000 cells/well and 2500 cells/well were seeded into 96-well plates for 48 h and 72 h treatments, respectively, and allowed to attach for 24 h. After attachment, the medium was replaced with variable concentrations of 2-AAPA in full growth medium. Cells were exposed to 2-AAPA for 48 h or 72 h followed by the MTT assay to determine cell survival rates. The IC50 values were calculated using a GraphPad Prism software.

### Protein *S*-glutathionylation detection

The protein *S*-glutathionylation induced by 2-AAPA in the TE-13 cells was detected using a detection kit from Cayman Chemical Company according to the manufacture’s instruction. Briefly, TE-13 cells were seeded at densities of 150,000 cells in a 96-well black wall plate. After a 24 h attachment at 37°C, TE-13 cells were treated with growth medium containing various concentrations of 2-AAPA for 1h. Washed the cells twice with PBS and then fixed them with 3.7% formaldehyde in PBS for 10 min at room temperature. Washed the cells once more with PBS and then blocked the protein free-thiol with 100 μL of PSSG Blocking Reagent for 30 min followed by twice wash with 1X Assay Buffer. 100 μL PSSG Reduction Reagent was added to each sample and incubated for 15 min at 37°C. Each sample was washed twice with 1X Assay Buffer and another 100 μL of PSSG Labeling Reagent was added to the samples followed by one-hour incubation. The samples were washed three times with 1X Assay Buffer and incubated with PSSG Detection Reagent II (FITC) at a 1:50 dilution in 1X Assay Buffer for 1 hour. The cells were washed twice with 1X Assay Buffer and the fluorescent images were captured using a Nikon Ti-S fluorescent microscope.

### Apoptotic assay by flow cytometry

TE-13 cells were seeded at densities of 300,000, 150,000 or 75,000 cells per well in 6-well plates for 24, 48 h and 72 h treatments, respectively. After a 24 h attachment at 37°C, TE-13 cells were treated with growth medium containing various concentrations of 2-AAPA. At the end of the treatments, the cells were collected by trypsinization. And then, Annexin V/PI staining was performed using BD Pharminge™ FITC-Annexin V Apoptosis Detection Kit I according to the manufacture’s instruction. The samples were analyzed with a Beckman Counter flow cytometer.

### Cell cycle analysis by flow cytometry

TE-13 cells were seeded and treated with 2-AAPA was described above for apoptotic assay. At the end of treatment, adherent cells were harvested and washed twice with ice-cold PBS. The cells of each sample were fixed with 70% ethanol in DPBS at 4°C overnight. The fixed cells were then centrifuged (700 ×g, 5 min) and washed with staining buffer. After the wash, the samples were centrifuged (700 ×g, 5 min), and the pellets were treated with 100 μL of RNase A (1 mg/mL) for 30 min at 37°C. After incubation, 900 μL of staining buffer was added to the samples to bring the volume to 1 mL followed by addition of 20 μL of propidium iodide (PI) (1 mg/mL). The sample was then incubated in the dark at room temperature for 30 min before being analyzed with a Beckman Counter flow cytometer.

### Western blot

TE-13 cells were seeded to 100 mm culture dishes at densities of 1.5 × 10^6^ and 1 × 10^6^ for 48 h and 72 treatments, respectively. The cells were allowed to attach for 24 h followed by treating in either growth medium with 0.025% DMSO as control or 2-AAPA (20, 35 and 50 μM). The TE-13 cells were collected by trypsinization and then lysed in RIPA lysis buffer. The protein concentrations were determined by bicinchoninic acid (BCA) method. Aliquots of 25-80 μg protein were fractionated in 10% and 12.5% sodium dodecylsulfate polyacrylamide gel (SDS-PAGE) under reducing conditions and then transferred to polyvinylidene fluoride (PVDF) membranes. After blocking with 5% non-fat milk, the membranes were probed with the appropriate dilution of primary antibodies followed by appropriate horseradishperoxidase (HRP) conjugated secondary antibody. The resulting conjugates were visualized using Pierce®ECL Plus kit in a ChemiDoc XPS system (Bio-Rad Laboratories, Inc., Hercules, CA, USA).

### Immunoprecipitation

Protein *S*-glutathionylation on specific proteins was determined by immunoprecipitation analysis. TE-13 cells treated with 80 μM of 2-AAPA or vehicle as control were lysed in immunoprecipitation buffer [50 mM Tris-HCl, 150 mM NaCl, 1% NP-40 (V/V), 2 mM EDTA] containing 1% protease inhibitor cocktail (Pierce, Thermo scientific) at 4 °C for 30 min. The lysate was then centrifuged at 15000 ×g for 10 min at 4°C. After that, samples (200 μg for each condition) were rotated with antibody at 4°C overnight. Protein A/G agarose was added to each samples and incubated for another 4 h at 4°C. Samples were then centrifuged at 500×g for 3 min at 4°C and washed 3 times with PBS under the same conditions, and subjected to immunoblotting analysis.

### ROS production

ROS production induced by 2-AAPA in the TE-13 cells was evaluated using the cell-permeative probe DCFH-DA. Upon entry into the cytoplasm, this probe is cleaved by cellular esterase and oxidized by ROS to yield green fluorescence. The ROS production in the TE-13 cells was determined by the method described earlier with minor modifications [71]. Briefly, attached exponentially growing TE-13 cells were treated in 10 μM DCFH-DA prepared in FBS-Free growth medium for 30 min at 37°C in dark. And the cells were washed with PBS three times and rapidly trypsinized and resuspended in full growth medium and then seeded into 96-well black well plates at a density of 25,000 cells/well. The cells were treated with 2-AAPA at concentrations of 20, 35 and 50 μM. The green fluorescence was measured at various time points at 480 nm (excitation) and 535 nm (emission) on a ThermoScientific VARIOSKAN FLASH microplate reader.

### Intracellular GSH and GSSG detection by LC-MS

Attached TE-13 cells were treated with 2-AAPA in culture dishes for various durations. The medium was collected, and the cells were rinsed with PBS and detached by trypsinization. The medium and the cell suspension were combined and centrifuged at 600 ×g for 5 min. Then the cell pellets were washed with cold PBS (1 mL × 2) and resuspended in 0.3 mL of 3% (w/v) sulfosalicylic acid. The cell suspension was sonicated over ice for 5 min and centrifuged at 16,000 ×g for another 10 min at 4°C. The supernatants of the cell lysates were diluted with 0.2% (w/v) formic acid for analysis of GSH and GSSG by following validated LC/MS method developed in this laboratory. The analysis was carried out on a ThermoFisher Ultimate 3000 RSLC system coupled with a Q-Exactive Orbitrap high resolution mass spectrometer, which is equipped with an electrospray ion source. Chromatographic separation of GSH and GSSG was achieved on a Thermo Scientific Hypersil GOLD C18 column (2.1×100 mm, 1.9 μm) with mobile phase A [aqueous solution with 0.2% (v/v) formic acid] and mobile phase B (acetonitrile with 0.2% (v/v) formic acid). Solvent B was first increased from 0% to 30% in 4 min, then to 80% in 2 min, and held at 80% for an additional 2 min. All flow rates were 0.2 mL/min. The injection volume was 15 μL. The electrospray ion source was operated in positive ionization mode and selected-ion monitoring (SIM) was performed to detect GSH, GSSG and p-aminobenzoic acid as the internal standard at the mass of m/z 307.08381 (M + H)^+^, m/z 612.15196 (M + H)^+^and m/z 138.05550 (M + H)^+^, respectively. The mass tolerance was set at 10 ppm.

### GR activity assay

The cell pellets obtained above were washed twice with ice-cold PBS. The cell pellets were resuspended in hypotonic phosphate buffer (1 mM, pH 7.4) containing 1 mM EDTA, and homogenized over ice with an Omni homogenizer. The homogenate was centrifuged at 150,000 ×g for 30 min at 4°C. The supernatant was collected and used to determine GR activity as described earlier [21]. Briefly, the assay mixture contained the supernatant (300 μL), BSA (1 mg/mL) and NADPH (0.2 mM). The enzymatic reaction was initiated by addition of GSSG (0.52 mM). GR activity was measured by the initial rates of disappearance of NADPH determined spectrophotometrically at 340 nm. Protein concentration of the homogenates was quantified by the BCA method.

### Indirect immunofluorescence microscopy

TE-13 cells were incubated with 2-AAPA at 37°C in a humidified atmosphere of 5% CO_2_ for 4 h. The immunofluorescence staining of microtubules were processed as described earlier [25]. Briefly, the TE-13 cells were fixed in 4% paraformaldehyde at room temperature for 1 h. The cells were washed 3 times with PBS and incubated with cell permeable solution (0.1% Na-citrate, 0.1% Triton-X-100 in PBS) at room temperature for 1 h. After incubation with the blocking solution (5% bovine serum albumin in PBS) overnight at 4°C, microtubules were visualized by incubating with a mouse monoclonal anti-α-tubulin-FITC (1:200) at 37°C for 2 h, and nuclei were stained with DAPI (1 μg/mL). Fluorescent images were taken with an Olympus Fluoview FV1200 microscope. Paclitaxel and vincristine sulfate were employed as positive controls for microtubule stabilization and depolymerization, respectively.

### Cell morphological change induced by 2-AAPA

Cultured TE-13 cells were treated with 40 and 60 μM 2-AAPA. Live cell images were taken by an Olympus CKX53 microscope with designed time intervals.

### Statistical analysis

Each experiment was performed in at least triplicate. Data were analyzed with a GraphPad Prism software. The two-tailed Student’s t-test was performed to interpret the differences between experimental and control groups. Significance in all experiments was considered at P < 0.05. Values were expressed as mean ± the standard deviation of the mean.

## References

[1] Peng X, Xue H, Lu L, Shi P, Wang J, Wang J (2017). Accumulated promoter methylation as a potential biomarker for esophageal cancer. Oncotarget.

[2] Torre LA, Bray F, Siegel RL, Ferlay J, Lortet-Tieulent J, Jemal A (2015). Global cancer statistics, 2012. CA Cancer J Clin.

[3] Ren QG, Yang SL, Hu JL, Li PD, Chen YS, Wang QS (2016). Evaluation of HO-1 expression, cellular ROS production, cellular proliferation and cellular apoptosis in human esophageal squamous cell carcinoma tumors and cell lines. Oncol Rep.

[4] Hsieh MS, Yang PW, Wong LF, Lee JM (2016). The AXL receptor tyrosine kinase is associated with adverse prognosis and distant metastasis in esophageal squamous cell carcinoma. Oncotarget.

[5] Lin Y, Totsuka Y, He Y, Kikuchi S, Qiao Y, Ueda J, Wei W, Inoue M, Tanaka H (2013). Epidemiology of esophageal cancer in Japan and China. J Epidemiol.

[6] Siegel R, Naishadham D, Jemal A (2012). Cancer statistics for Hispanics/Latinos, 2012. CA Cancer J Clin.

[7] Li CC, Chen CY, Chien CR (2016). Comparative effectiveness of image-guided radiotherapy for non-operated localized esophageal squamous cell carcinoma patients receiving concurrent chemoradiotherapy: a population-based propensity score matched analysis. Oncotarget.

[8] Liu T, Li R, Zhao H, Deng J, Long Y, Shuai MT, Li Q, Gu H, Chen YQ, Leng AM (2016). eIF4E promotes tumorigenesis and modulates chemosensitivity to cisplatin in esophageal squamous cell carcinoma. Oncotarget.

[9] Gaur P, Kim MP, Dunkin BJ (2014). Esophageal cancer: recent advances in screening, targeted therapy, and management. J Carcinog.

[10] Hua P, Sun M, Zhang G, Zhang Y, Song G, Liu Z, Li X, Zhang X, Li B (2016). Costunolide induces apoptosis through generation of ROS and activation of P53 in human esophageal cancer Eca-109 cells. J Biochem Mol Toxicol.

[11] Zhang X, Chen M, Zou P, Kanchana K, Weng Q, Chen W, Zhong P, Ji J, Zhou H, He L, Liang G (2015). Curcumin analog WZ35 induced cell death via ROS-dependent ER stress and G2/M cell cycle arrest in human prostate cancer cells. BMC Cancer.

[12] You BR, Park WH (2016). Auranofin induces mesothelioma cell death through oxidative stress and GSH depletion. Oncol Rep.

[13] Trachootham D, Lu W, Ogasawara MA, Nilsa RD, Huang P (2008). Redox regulation of cell survival. Antioxid Redox Signal.

[14] Glasauer A, Chandel NS (2014). Targeting antioxidants for cancer therapy. Biochem Pharmacol.

[15] Weinberg F, Chandel NS (2009). Reactive oxygen species-dependent signaling regulates cancer. Cell Mol Life Sci.

[16] Subramani R, Gonzalez E, Arumugam A, Nandy S, Gonzalez V, Medel J, Camacho F, Ortega A, Bonkoungou S, Narayan M, Dwivedi A, Lakshmanaswamy R (2016). Nimbolide inhibits pancreatic cancer growth and metastasis through ROS-mediated apoptosis and inhibition of epithelial-to-mesenchymal transition. Sci Rep.

[17] Tamma G, Valenti G (2016). Evaluating the oxidative stress in renal diseases: what is the role for s-glutathionylation?. Antioxid Redox Signal.

[18] Zhang H, Yang J, Wu S, Gong W, Chen C, Perrett S (2016). Glutathionylation of the bacterial Hsp70 chaperone dnak provides a link between oxidative stress and the heat shock response. J Biol Chem.

[19] Mieyal JJ, Chock PB (2012). Posttranslational modification of cysteine in redox signaling and oxidative stress: focus on s-glutathionylation. Antioxid Redox Signal.

[20] Li Q, Zhan M, Chen W, Zhao B, Yang K, Yang J, Yi J, Huang Q, Mohan M, Hou Z, Wang J (2016). Phenylethyl isothiocyanate reverses cisplatin resistance in biliary tract cancer cells via glutathionylation-dependent degradation of Mcl-1. Oncotarget.

[21] Seefeldt T, Zhao Y, Chen W, Raza AS, Carlson L, Herman J, Stoebner A, Hanson S, Foll R, Guan X (2009). Characterization of a novel dithiocarbamate glutathione reductase inhibitor and its use as a tool to modulate intracellular glutathione. J Biol Chem.

[22] Zhao Y, Seefeldt T, Chen W, Wang X, Matthees D, Hu Y, Guan X (2009). Effects of glutathione reductase inhibition on cellular thiol redox state and related systems. Arch Biochem Biophys.

[23] Chen W, Zhao Y, Seefeldt T, Guan X (2008). Determination of thiols and disulfides via HPLC quantification of 5-thio-2-nitrobenzoic acid. J Pharm Biomed Anal.

[24] Xie J, Potter A, Xie W, Lynch C, Seefeldt T (2014). Evaluation of a dithiocarbamate derivative as a model of thiol oxidative stress in H9c2 rat cardiomyocytes. Free Radic Biol Med.

[25] Chen W, Seefeldt T, Young A, Zhang X, Zhao Y, Ruffolo J, Kaushik RS, Guan X (2012). Microtubule S-glutathionylation as a potential approach for antimitotic agents. BMC Cancer.

[26] Couto N, Wood J, Barber J (2016). The role of glutathione reductase and related enzymes on cellular redox homoeostasis network. Free Radic Biol Med.

[27] Xiong Y, Uys JD, Tew KD, Townsend DM (2011). S-glutathionylation: from molecular mechanisms to health outcomes. Antioxid Redox Signal.

[28] Ashwaq AS, Al-Qubaisi MS, Rasedee A, Abdul AB, Taufiq-Yap YH, Yeap SK (2016). Inducing G2/M Cell Cycle arrest and apoptosis through generation reactive oxygen species (ROS)-mediated mitochondria pathway in HT-29 cells by dentatin (DEN) and dentatin incorporated in hydroxypropyl-beta-cyclodextrin (DEN-HPbetaCD). International journal of molecular sciences.

[29] Bodas M, Van Westphal C, Carpenter-Thompson R, K Mohanty D, Vij N (2016). Nicotine exposure induces bronchial epithelial cell apoptosis and senescence via ROS mediated autophagy-impairment. Free Radic Biol Med.

[30] Park H, Kim CH, Jeong JH, Park M, Kim KS (2016). GDF15 contributes to radiation-induced senescence through the ROS-mediated p16 pathway in human endothelial cells. Oncotarget.

[31] Baek MW, Cho HS, Kim SH, Kim WJ, Jung JY (2017). Ascorbic acid induces necrosis in human laryngeal squamous cell carcinoma via ROS, PKC, and calcium signaling. J Cell Physiol.

[32] Trachootham D, Alexandre J, Huang P (2009). Targeting cancer cells by ROS-mediated mechanisms: a radical therapeutic approach?. Nat Rev Drug Discov.

[33] Jayakumar S, Patwardhan RS, Pal D, Sharma D, Sandur SK (2016). Dimethoxycurcumin, a metabolically stable analogue of curcumin enhances the radiosensitivity of cancer cells: possible involvement of ROS and thioredoxin reductase. Biochem Biophys Res Commun.

[34] Prasad S, Gupta SC, Tyagi AK (2017). Reactive oxygen species (ROS) and cancer: role of antioxidative nutraceuticals. Cancer Lett.

[35] Panieri E, Santoro MM (2016). ROS homeostasis and metabolism: a dangerous liason in cancer cells. Cell Death Dis.

[36] Jung SH, Kim SM, Lee CE (2016). Mechanism of suppressors of cytokine signaling 1 inhibition of epithelial-mesenchymal transition signaling through ROS regulation in colon cancer cells: suppression of Src leading to thioredoxin up-regulation. Oncotarget.

[37] Zong L, Li J, Chen X, Chen K, Li W, Li X, Zhang L, Duan W, Lei J, Xu Q, Shan T, Ma Q, Sun H (2016). Lipoxin A4 attenuates cell invasion by inhibiting ROS/ERK/MMP pathway in pancreatic cancer. Oxid Med Cell Longev.

[38] Li P, Zhang D, Shen L, Dong K, Wu M, Ou Z, Shi D (2016). Redox homeostasis protects mitochondria through accelerating ROS conversion to enhance hypoxia resistance in cancer cells. Sci Rep.

[39] Perry G, Raina AK, Nunomura A, Wataya T, Sayre LM, Smith MA (2000). How important is oxidative damage? Lessons from Alzheimer’s disease. Free Radic Biol Med.

[40] Pelicano H, Carney D, Huang P (2004). ROS stress in cancer cells and therapeutic implications. Drug Resist Updat.

[41] Shan F, Shao Z, Jiang S, Cheng Z (2016). Erlotinib induces the human non-small-cell lung cancer cells apoptosis via activating ROS-dependent JNK pathways. Cancer Med.

[42] Taha MM, Sheikh BY, Salim LZ, Mohan S, Khan A, Kamalidehghan B, Ahmadipour F, Abdelwahab SI (2016). Thymoquinone induces apoptosis and increase ROS in ovarian cancer cell line. Cell Mol Biol.

[43] Sung B, Ravindran J, Prasad S, Pandey MK, Aggarwal BB (2016). Gossypol induces death receptor-5 through activation of ROS-ERK-CHOP pathway and sensitizes colon cancer cells to TRAIL. J Biol Chem.

[44] Du ZX, Zhang HY, Meng X, Guan Y, Wang HQ (2009). Role of oxidative stress and intracellular glutathione in the sensitivity to apoptosis induced by proteasome inhibitor in thyroid cancer cells. BMC Cancer.

[45] Thangamani S, Eldesouky HE, Mohammad H, Pascuzzi PE, Avramova L, Hazbun TR, Seleem MN (2016). Ebselen exerts antifungal activity by regulating glutathione (GSH) and reactive oxygen species (ROS) production in fungal cells. Biochim Biophys Acta.

[46] Hua P, Sun M, Zhang G, Zhang Y, Song G, Liu Z, Li X, Zhang X, Li B (2016). Costunolide induces apoptosis through generation of ROS and activation of P53 in human esophageal cancer eca-109 Cells. J Biochem Mol Toxicol.

[47] Zhang C, Liu K, Yao K, Reddy K, Zhang Y, Fu Y, Yang G, Zykova TA, Shin SH, Li H, Ryu J, Jiang YN, Yin X (2015). HOI-02 induces apoptosis and G2-M arrest in esophageal cancer mediated by ROS. Cell Death Dis.

[48] Vousden KH, Lane DP (2007). p53 in health and disease. Nat Rev Mol Cell Biol.

[49] Stewart N, Hicks GG, Paraskevas F, Mowat M (1995). Evidence for a second cell cycle block at G2/M by p53. Oncogene.

[50] Agarwal ML, Agarwal A, Taylor WR, Stark GR (1995). p53 controls both the G2/M and the G1 cell cycle checkpoints and mediates reversible growth arrest in human fibroblasts. Proc Natl Acad Sci U S A.

[51] Sugrue MM, Shin DY, Lee SW, Aaronson SA (1997). Wild-type p53 triggers a rapid senescence program in human tumor cells lacking functional p53. Proc Natl Acad Sci U S A.

[52] Hermeking H, Lengauer C, Polyak K, He TC, Zhang L, Thiagalingam S, Kinzler KW, Vogelstein B (1997). 14-3-3sigma is a p53-regulated inhibitor of G2/M progression. Mol Cell.

[53] Innocente SA, Abrahamson JL, Cogswell JP, Lee JM (1999). p53 regulates a G2 checkpoint through cyclin B1. Proc Natl Acad Sci U S A.

[54] Fischer M, Quaas M, Steiner L, Engeland K (2016). The p53-p21-DREAM-CDE/CHR pathway regulates G2/M cell cycle genes. Nucleic Acids Res.

[55] Umezawa Y, Kurosu T, Akiyama H, Wu N, Nogami A, Nagao T, Miura O (2016). Down regulation of Chk1 by p53 plays a role in synergistic induction of apoptosis by chemotherapeutics and inhibitors for Jak2 or BCR/ABL in hematopoietic cells. Oncotarget.

[56] el-Deiry WS, Tokino T, Velculescu VE, Levy DB, Parsons R, Trent JM, Lin D, Mercer WE, Kinzler KW, Vogelstein B (1993). WAF1, a potential mediator of p53 tumor suppression. Cell.

[57] el-Deiry WS, Harper JW, O’Connor PM, Velculescu VE, Canman CE, Jackman J, Pietenpol JA, Burrell M, Hill DE, Wang Y Wilman KG, Edward Mercer W, Kastan MB (1994). WAF1/CIP1 is induced in p53-mediated G1 arrest and apoptosis. Cancer research.

[58] Kang N, Jian JF, Cao SJ, Zhang Q, Mao YW, Huang YY, Peng YF, Qiu F, Gao XM (2016). Physalin A induces G2/M phase cell cycle arrest in human non-small cell lung cancer cells: involvement of the p38 MAPK/ROS pathway. Mol Cell Biochem.

[59] Walsh S, Margolis SS, Kornbluth S (2003). Phosphorylation of the cyclin b1 cytoplasmic retention sequence by mitogen-activated protein kinase and Plx. Mol Cancer Res.

[60] St Clair S, Giono L, Varmeh-Ziaie S, Resnick-Silverman L, Liu WJ, Padi A, Dastidar J, DaCosta A, Mattia M, Manfredi JJ (2004). DNA damage-induced downregulation of Cdc25C is mediated by p53 via two independent mechanisms: one involves direct binding to the cdc25C promoter. Mol Cell.

[61] Pyo CW, Choi JH, Oh SM, Choi SY (2013). Oxidative stress-induced cyclin D1 depletion and its role in cell cycle processing. Biochim Biophys Acta.

[62] Niwa T (2007). Protein glutathionylation and oxidative stress. J Chromatogr B Analyt Technol Biomed Life Sci.

[63] Popov D (2014). Protein S-glutathionylation: from current basics to targeted modifications. Arch Physiol Biochem.

[64] Lind C, Gerdes R, Hamnell Y, Schuppe-Koistinen I, von Lowenhielm HB, Holmgren A, Cotgreave IA (2002). Identification of S-glutathionylated cellular proteins during oxidative stress and constitutive metabolism by affinity purification and proteomic analysis. Arch Biochem Biophys.

[65] Checconi P, Salzano S, Bowler L, Mullen L, Mengozzi M, Hanschmann EM, Lillig CH, Sgarbanti R, Panella S, Nencioni L, Palamara AT, Ghezzi P (2015). Redox proteomics of the inflammatory secretome identifies a common set of redoxins and other glutathionylated proteins released in inflammation, influenza virus infection and oxidative stress. PLoS One.

[66] Salzano S, Checconi P, Hanschmann EM, Lillig CH, Bowler LD, Chan P, Vaudry D, Mengozzi M, Coppo L, Sacre S, Atkuri KR, Sahaf B, Herzenberg LA (2014). Linkage of inflammation and oxidative stress via release of glutathionylated peroxiredoxin-2, which acts as a danger signal. Proc Natl Acad Sci U S A.

[67] Andrei D, Maciag AE, Chakrapani H, Citro ML, Keefer LK, Saavedra JE (2008). Aryl bis(diazeniumdiolates): potent inducers of S-glutathionylation of cellular proteins and their *in vitro* antiproliferative activities. J Med Chem.

[68] Armeni T, Ercolani L, Urbanelli L, Magini A, Magherini F, Pugnaloni A, Piva F, Modesti A, Emiliani C, Principato G (2012). Cellular redox imbalance and changes of protein S-glutathionylation patterns are associated with senescence induced by oncogenic H-ras. PLoS One.

[69] Ghezzi P, Di Simplicio P (2007). Glutathionylation pathways in drug response. Curr Opin Pharmacol.

[70] Sadhu SS, Callegari E, Zhao Y, Guan X, Seefeldt T (2013). Evaluation of a dithiocarbamate derivative as an inhibitor of human glutaredoxin-1. J Enzyme Inhib Med Chem.

[71] Chen W, Jiang Z, Zhang X, Feng J, Ling Y (2015). Nacetyl-S-(p-chlorophenylcarbamoyl)cysteine induces mitochondrial-mediated apoptosis and suppresses migration in melanoma cells. Oncol Rep.

